# *In vivo* bioluminescence imaging of vascular remodeling after stroke

**DOI:** 10.3389/fncel.2014.00274

**Published:** 2014-09-05

**Authors:** Joanna M. Adamczak, Gabriele Schneider, Melanie Nelles, Ivo Que, Ernst Suidgeest, Louise van der Weerd, Clemens Löwik, Mathias Hoehn

**Affiliations:** ^1^In-vivo-NMR Laboratory, Max-Planck-Institute for Neurological ResearchCologne, Germany; ^2^Department of Endocrinology, Leiden University Medical CenterLeiden, Netherlands; ^3^Department of Radiology, Leiden University Medical CenterLeiden, Netherlands; ^4^Department of Human Genetics, Leiden University Medical CenterLeiden, Netherlands

**Keywords:** VEGFR2, flk-1, cerebral ischemia, angiogenesis, vessel density

## Abstract

Thrombolysis remains the only beneficial therapy for ischemic stroke, but is restricted to a short therapeutic window following the infarct. Currently research is focusing on spontaneous regenerative processes during the sub-acute and chronic phase. Angiogenesis, the formation of new blood vessels from pre-existing ones, was observed in stroke patients, correlates with longer survival and positively affects the formation of new neurons. Angiogenesis takes place in the border zones of the infarct, but further insight into the temporal profile is needed to fully apprehend its therapeutic potential and its relevance for neurogenesis and functional recovery. Angiogenesis is a multistep process, involving extracellular matrix degradation, endothelial cell proliferation, and, finally, new vessel formation. Interaction between vascular endothelial growth factor and its receptor 2 (VEGFR2) plays a central role in these angiogenic signaling cascades. In the present study we investigated non-invasively the dynamics of VEGFR2 expression following cerebral ischemia in a mouse model of middle cerebral artery occlusion (MCAO). We used a transgenic mouse expressing firefly luciferase under the control of the VEGFR2 promotor to non-invasively elucidate the temporal profile of VEGFR2 expression after stroke as a biomarker for VEGF/VEGFR2 signaling. We measured each animal repetitively up to 2 weeks after stroke and found increased VEGFR2 expression starting 3 days after the insult with peak values at 7 days. These were paralleled by increased VEGFR2 protein levels and increased vascular volume in peri-infarct areas at 14 days after the infarct, indicating that signaling via VEGFR2 leads to successful vascular remodeling. This study describes VEGFR2-related signaling is active at least up to 2 weeks after the infarct and results in increased vascular volume. Further, this study presents a novel strategy for the non-invasive evaluation of angiogenesis-based therapies.

## Introduction

Angiogenesis, the formation of new blood vessels from pre-existing ones, is recognized as a potential new therapeutic target in ischemic stroke (Slevin et al., 2006; Navaratna et al., 2009; Shibuya, 2009). Increased vascularization in areas surrounding the infarct has been observed in human (Krupinski et al., 1994; Szpak et al., 1999) as well as in animal brain tissue (Beck et al., 2000; Marti et al., 2000; Hayashi et al., 2003; Thored et al., 2007; Li et al., 2011) and is associated with improved functionality (Krupinski et al., 1994; Wang et al., 2006; Lee et al., 2007b; Reitmeir et al., 2012). Furthermore, angiogenesis is closely linked to neurogenesis and has shown positive effects on neuronal cell formation, migration and maturation (Taguchi et al., 2004). These results support the hypothesis, that angiogenesis after stroke is therapeutically advantageous.

The absence of adequate blood supply caused by the blockage of a cerebral artery leads to tissue hypoxia, which triggers the angiogenic response (Marti et al., 2000; Beck and Plate, 2009). Hypoxia inducible factor (HIF) 1α is stabilized under hypoxic conditions and dimerizes with HIF1β to form a transcription factor, which binds to the hypoxia responsible element in the promotor region of several hypoxia inducible cytokines and growth factors (Ferrara et al., 2003; Hayashi et al., 2006). The most potent angiogenic factor is the vascular endothelial growth factor (VEGF), which exerts its effect through its main receptor, i.e., vascular endothelial growth factor receptor 2 (VEGFR2; Ferrara et al., 2003; Koch et al., 2011). Activation of the VEGFR2 results in endothelial cell proliferation, migration and differentiation (Ferrara et al., 2003; Hayashi et al., 2006). Therefore, VEGFR2 plays a key role in adult angiogenesis and represents a molecular biomarker for angiogenic signaling in tissue. In a mouse model of middle cerebral artery occlusion (MCAO), VEGFR2 was upregulated as early as 1 h poststroke, continued to increase for up to 1 week and decreased thereafter (Marti et al., 2000; Hayashi et al., 2003; Cai et al., 2009). Poststroke VEGFR2 expression was observed in neuronal and endothelial cells—neuronal VEGFR2 expression was early and transient, while its expression on endothelial cells persisted for prolonged time after the ischemic insult (Hayashi et al., 2003). Although poststroke VEGFR2 induction is not restricted to endothelial cells, its sub-acute and chronic expression is pre-dominently on endothelial cells. Furthermore, poststroke angiogenesis is VEGF/VEGFR2 signaling dependent, as inhibition of the VEGFR2 receptor resulted in decreased vascular regeneration and reduced regional cerebral blood flow (Li et al., 2011). The time profile of VEGFR2 expression after stroke is mainly based upon invasive methods like immunohistochemistry (IHC), Western blot (WB) and mRNA analysis. Up to date, only one study approached the non-invasive tracking of VEGFR2 expression as a correlate for angiogenesis after stroke (Cai et al., 2009). Using a PET-tracer for VEGF receptors Cai et al. (2009) found increased VEGFR2 expression in the ischemic hemisphere of rats until 16 days after stroke, and it subsequently decreased to almost normal levels at 23 days post-stroke. However, the tracer detected both, VEGFR2 and VEGFR1.

In the following study we utilized the VEGFR2 as a biomarker for the molecular regulation of angiogenic remodeling after stroke. We used the non-invasive imaging technique of bioluminescence to monitor the dynamic changes in VEGFR2 expression in a VEGFR2-luc reporter mouse with VEGFR2-specific photon emission (PE). We observed the temporal dynamics of VEGFR2 expression up to 2 weeks after stroke and validated the elevated VEGFR2 levels in the ischemic hemisphere by Western blotting of the receptor. Subsequently we evaluated the effect of increased VEGFR2 expression on vessel density by quantifying changes in vascular volume on immunohistological sections.

## Materials and methods

### Animal model

All animal experiments were conducted according to the guidelines laid out in the German Animal Welfare Act, in accordance with the European Council Directive 2010/63/EU, and were approved by the Landesamt für Natur, Umwelt und Verbraucherschutz North Rhine-Westphalia, reference number 84-02.04.2011.A123, as well as by the bioethics committee from Leiden University Medical Center, Leiden, The Netherlands, reference number 10215. A transgenic knock-in mouse model (Lyons et al., 2005) which expresses firefly luciferase under the control of the VEGFR2 promotor was used for all experiments. The animals were kept under *ad libitum* supply of food and water in a 12 h/12 h day and night cycle. All measurements and surgical interventions were performed under isoflurane anesthesia.

### Experimental groups

**Figure 1:**
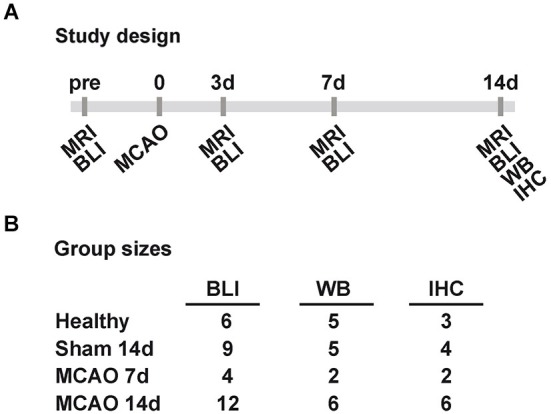
**Study design and group sizes. (A)** Sham and MCAO animals were measured before (pre), and 3, 7, 14 days (3d, 7d, 14d) after 30 min MCAO with MRI and BLI. At 7d and 14d post MCAO tissue was collected for WB and IHC. **(B)** The displayed final group sizes as used per method and group. Excluded animals are not listed. (MCAO: middle cerebral artery occlusion, MRI: magnetic resonance imaging, BLI: bioluminescence imaging, WB: western blot, IHC: immunohistochemistry).

In total 39 male VEGFR2-luc knock-in mice (7–13 weeks old) were randomly assigned into groups of different survival times and different post mortem tissue processing. Six healthy control animals were measured with bioluminescence at three different days for the assessment of inter- and intra-animal stability of bioluminescence kinetics from the brain. Twelve animals received a sham surgery of which three had to be excluded due to death (*n* = 1) and spontaneous hyperintense brain areas on T2-weighted MR images (*n* = 2). Eighteen mice received a 30 min occlusion of the right middle cerebral artery of which two were excluded due to lack of stroke (*n* = 1) and strong weight loss (*n* = 1). Middle cerebral artery occlusion and sham animals were imaged 3 to 7 days before surgery (baseline acquisition) and 3, 7 and 14 days post surgery. Each bioluminescence imaging (BLI) session was directly followed by a magnetic resonance imaging (MRI) acquisition of T2 maps. Between day 3 and day 7 post surgery, sham and stroke animals received injections of 5-bromo-2′-deoxyuridine (BrdU, Sigma Aldrich, Taufkirchen, Germany) twice daily (50 mg/kg). At the end time point brain tissue was collected for either Western blots or IHC. An overview of the study design and final group sizes is presented in Figure 1.

### Middle cerebral artery occlusion

The ischemic lesion was induced by transient occlusion of the right middle cerebral artery (MCAO), using the intraluminal filament model adapted from rat. The specific surgical method used in this study equals previously described MCAO in mice (Bahmani et al., 2011). Mice were anesthetized with 1–2% isoflurane in a 30/70 oxygen/air mixture and received a subcutaneous injection of 4 mg/kg buprenorphin (Temgesic, Merck, Darmstadt, Germany) for analgesia. A neck incision exposed the common carotid artery (CCA) and a silicon rubber-coated filament with a tip diameter of 170 μm (7017PK5Re, Doccol Corporation, Sharon, MA USA) was introduced into its lumen. The filament was advanced through the internal carotid artery (ICA) until it blocked the blood flow to the middle cerebral artery. Animals were allowed to recover under a red light lamp during the occlusion period. After 30 min of occlusion, animals were re-anesthetized and reperfusion was initiated by filament removal. The CCA was permanently ligated. Sham surgery involved the partial introduction of a filament into the CCA without blocking the blood flow to the MCA. Animals were also recovered for 30 min and re-anesthetized for filament removal. The CCA was also ligated permanently. Following MCAO surgery, all animals received s.c. injections of 1 ml NaCl twice daily until the body weight stabilized.

### Bioluminescence imaging

**Figure 2:**
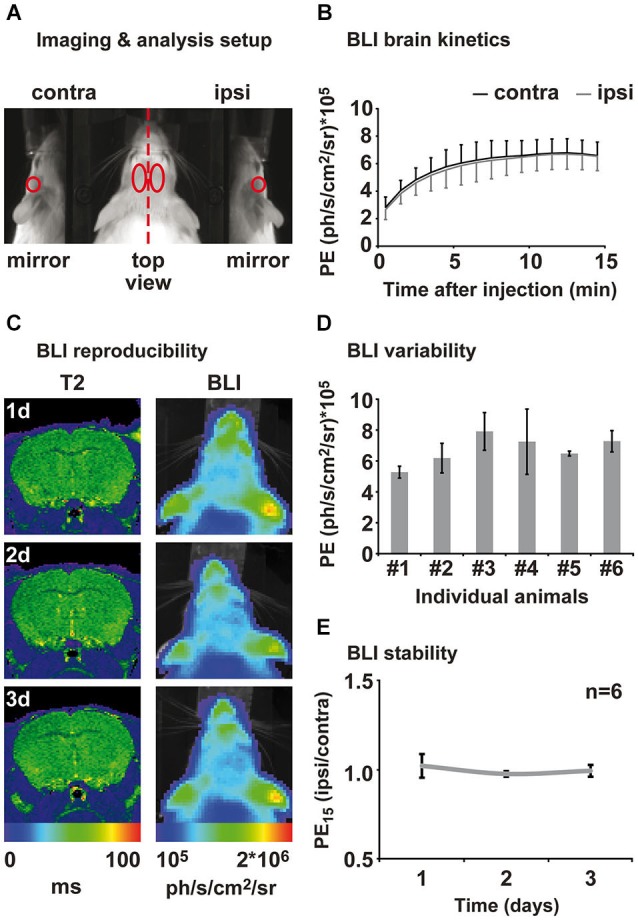
**Assessment of brain kinetics and stability of the BLI signal from healthy animals. (A)** Animals were placed in a prone position on an elevated bar between two 45° mirrors. BLI was evaluated in four different regions of interest: ipsi- and contralateral top view, ipsi- and contra-lateral mirror (side) view. **(B)** Measuring PE directly after luciferin injection reveals brain specific inflow kinetics. An inflow phase between 1–10 min can be distinguished from a plateau phase of maximal PE between 12 and 15 min after injection. Emission from ipsi- and contralateral hemisphere was consistently of same magnitude. **(C)** Healthy control animals were measured repetitively for the assessment of the inter- and intra-animal variability of the BLI signal. MRI and BLI images of an exemplary healthy animal show an intact brain and stable intensity of PE over time. MRI is shown as coronal brain section, BLI is shown as horizontal planar projection of the PE onto the mouse photograph. **(D)** Three-fold measurement of each healthy animal revealed intra-individual variability of 3–30%. Inter-animal variability was around 20% to 45%. **(E)** Normalization of PE from ipsi- to contralateral hemisphere eliminates intra- and inter-animal variability and results in stable bioluminescence over time. Data is presented as mean ± standard deviation. (PE: photon emission, PE15: photon emission of the 15th minute after luciferin injection).

One day prior to the first BLI session, mice were anesthetized in 2% isoflurane and the fur on the head was shaved to allow better photon penetration. It was not necessary to shave the animals a second time during the study. Photon emission was captured using the IVIS 100 (Perkin-Elmer, Waltham, MA, USA) equipped with a mirror system (prototype, Perkin-Elmer) consisting of two mirrors at a 45° angle to the basis (Figure 2A). Mice were individually anesthetized in 2% isoflurane and subsequently injected i.p. with 150 mg/kg click beetle luciferin (Promega, Madison, WI, USA) (stock solution 20 mg/ml). The acquisition of PE was directly started after luciferin injection with Living Image software (binning = 4, aperture = 1; Perkin-Elmer). Fifteen consecutive measurements of 1 min duration were performed in order to capture the inflow kinetics. Photon emission was analyzed for different regions of interest (ROIs; Figure 2A). Regions of interest were kept constant in size and were positioned on the photographic image using anatomical landmarks for guidance (ears, eyes; compare Figure 2A). Inflow kinetics and PE of the 15th min (PE15) after injection were extracted for each ROI. PE15 values from the “ischemic hemisphere” ROI and the “mirror ischemic” ROI were subsequently divided by the “intact hemisphere” and “mirror intact” ROI, respectively, in order to receive a quantitative ratio of change relative to the intact hemisphere. All values are displayed as mean ± standard deviation.

### Magnetic resonance imaging

Experiments were performed on a 7 Tesla Bruker Pharmascan 70/16 (Bruker Biospin, Ettlingen, Germany) with a 16 cm horizontal bore magnet and a 9 cm (inner diameter) shielded gradient, a maximum gradient strength of 300 mT/m, and a 23-mm birdcage transmit-receive RF coil (Bruker Biospin, Ettlingen, Germany). A multi-slice multi-echo (MSME) sequence (TR/TE = 4000 ms /11 ms, 16 echoes, 8 slices, slice thickness 1 mm, FOV 1.5 × 1.5 cm, matrix 128 × 128, resolution 117 × 117 μm) was measured for T2 evaluation and visualization of lesion location. T2 maps were calculated with IDL software (Exelis Visual Information Soution, Boulder, CO, USA).

### Western blotting

Animals were deeply anesthetized and killed by cervical dislocation. Brains were removed quickly and placed in ice cold phosphate buffered saline (PBS). Left and right cortex, as well as left and right striatum were dissected and directly frozen on dried ice. Tissue was stored at −80°C until further processing. Tissue was lysated in cell lysis buffer (#9803, Cell signaling Technology, Beverly, MA, USA) and treated with protease inhibitor complete Mini (CatNo 04693159001, Roche Applied Science, Indianapolis, Indiana, USA) supplemented with phenylmethylsulphonyl fluoride (PMSF, P6726, Sigma-Aldrich, Taufkirchen, Germany). Protein concentration was determined using the BCA Protein Assay Kit (Pierce, Rockford, IL, USA). For each sample equal amounts of protein were electrophoresed through 8–16% SDS–PAGE gel (Invitrogen, Life Technologies, Darmstadt, Germany) and subsequently electrotransferred to nitrocellulose membranes (ProTran, Whatman, Kent, UK). Membranes were probed with the primary antibodies for VEGFR2 (1:500; #2479, Cell Signaling Technology, Beverly, MA, USA), and β-actin (1:5000; MP Biomedical, Solon, Ohio, USA) overnight at 4°C. For detection, horseradish peroxidase-conjugated secondary antibodies were used (1:3000 for β-actin, 1:800 for VEGFR2) followed by enhanced chemiluminescence development with Amersham ECL Western Blotting Detection Reagents (GE Healthcare, Buckinghamshire, UK). Results were analyzed using ImageJ software^1^ (NIH). Regions of interest with constant size were positioned over each protein band and the integrated density was quantified followed by background subtraction and normalization to the β-actin signal. Data from the ischemic/sham hemisphere was then normalized to the intact hemisphere and displayed as mean ± standard deviation.

### Immunohistochemistry

Animals were deeply anesthetized and transcardially perfused with ice cold PBS followed by 20 ml 4% paraformaldehyde. Subsequently, brains were removed and shock frozen in −40°C methylbutane (Sigma-Aldrich, Taufkirchen, Germany). Brain tissue was stored at −80°C until further processing. Brain sections of 10 μm thickness were cut on the cryostat (Leica Microsystems, Wetzlar, Germany) and stored at −20°C. Sections for BrdU staining were pretreated with 2N hydrochloric acid for 2 h at room temperature. Prior to immunostaining, sections were pre-incubated at room temperature in 5% normal serum and 0.25% Triton X-100, in KPBS for 45 min. Primary antibodies were incubated overnight at 4°C. The following primary antibodies were used for double staining: anti-laminin (1:100, ab11575, Abcam, Cambridge, UK), anti-GFAP (1:200, G3898, Abcam Cambridge, UK), biotinylated *Solanum tuberosum* (potato) lectin (1:100, B-1165, Vector Laboratories, Burlingame, CA, USA), anti-BrdU (1:100, ab6326, Abcam, Cambridge, UK). Secondary antibodies were applied for 2 h at room temperature. A biotin-conjugated secondary antibody (1:200, Vector Laboratories, Burlingame, USA) was used with Alexa 488-conjugated streptavidin (1:200, Molecular Probes, Invitrogen, Life Technologies, Darmstadt, Germany). Cy2 and Cy3 (1:200, Jackson Immuno Research, West Grove, PA, USA) were used as complementary secondary antibodies for double staining, and Hoechst 33342 (1:1000 Invitrogen, Carlsbad, USA) was added during final incubation with secondary antibodies for nuclear staining. Slides were coverslipped with mounting medium (Entellan, Merck, Darmstadt, Germany). Z-stacks of BrdU/lectin positive cells were acquired with a confocal microscope (Leica TCS SP8, Leica Microsystems, Wetzlar, Germany). Laminin/GFAP double staining was used for vascular volume estimation. Microscopic images of whole brain sections were acquired at 4× magnification with a fluorescent microscope (BZ-9000 Keyence, Osaka, Japan). Areas of interest were defined inside the glial scar (“cortex core”, “striatum core”) and outside the glial scar (“cortex peri”, “striatum peri”). From each area of interest three images were taken at 20× magnification from the ischemic side and from corresponding homotopic areas within the intact hemisphere, while holding exposure time constant. Using the Keyence microscope processing software, the area of staining was quantified in these ROIs by fixed thresholding and constant cut-off of clusters of small size (<100 pixels), which were regarded as unspecific dirt and were eliminated from the selection. Subsequently, the area covered by the staining was calculated for each image and a ratio was made to the corresponding image of the intact hemisphere. This procedure was chosen to conservatively control for differences in staining intensities between animal sections, although the staining was highly reproducible and in particular even across sections. A mean was calculated for each region for each animal. Subsequently, a group mean was calculated for each region. Data is presented as group mean ± standard deviation.

### Statistics

Statistical analysis was performed with SPSS version 15 (IBM SPSS statistics, Ehningen, Germany). *In vivo* BLI data was tested for significant changes using a repeated measures analysis of variance (RM-ANOVA) with Bonferroni corrected *post hoc* comparisons. Western blot data was tested for significant changes by one way analysis of variance (one way ANOVA) with least significant difference *post hoc* comparisons between groups. Using an independent one-tailed Student’s *t*-test we tested vascular volume for significant changes between the stroke and the sham group. We performed bivariate correlation analysis between bioluminescence and Western blot data as well as between bioluminescence and vascular volume data with Spearman’s *ρ* correlation coefficient. A *p*-value ≤ 0.05 was considered to be statistically significant.

## Results

### Inter- and intra-animal stability and kinetics of the bioluminescence signal

We characterized the PE kinetics from the brain of six healthy transgenic mice expressing firefly luciferase under the control of the VEGFR2 promotor using the ROIs depicted in Figure 2A. All animals showed increasing PE from the brain between 1 to 10 min after luciferin injection. Photon emission reached a maximum between 10 and 13 min and remained on this level in a plateau phase until the end of the measurement at 15 min post injection (Figure 2B). Photon emission kinetics and intensity from the right (ipsilateral) hemisphere were equal to the left (contralateral) hemisphere for each individual animal (Figure 2B). We therefore continued to evaluate the PE of the 15th minute after luciferin injection (PE_15_), which represents maximum PE. Healthy animals were measured at three consecutive days for the assessment of inter- and intra-animal stability. Repetitive MRI on the healthy subjects reveals stable T2 values between time points and equality of both hemispheres. Corresponding BLI of this exemplary animal, presented as color-coded PE_15_, confirms reproducibly equal VEGFR2 baseline expression in both hemispheres, as well as a stable PE_15_ over time (Figure 2C). Nevertheless, repeated measurement of the same animal resulted in PE_15_ variations from 3 to 30% of the mean and absolute signal intensity variation between animals was between 20 to 45% of the mean (Figure 2D). In order to investigate BLI changes over time, we corrected for inter- and intra-individual variation by normalizing the ischemic (ipsilateral) to the intact (contralateral) hemisphere. This analysis strategy shows stable equal VEGFR2 expression in healthy subjects over time (Figure 2E), thus allowing for reproducible and stable assessment of the hemispheric differences in VEGFR2 expression.

### Cerebral ischemia

**Figure 3:**
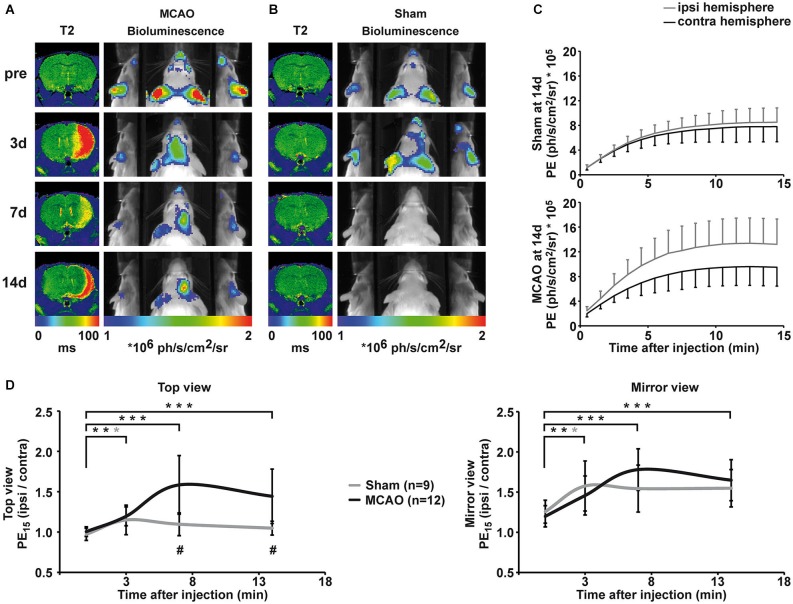
**Qualitative and quantitative evaluation of BLI changes after sham and MCAO surgery. (A)** Representative longitudinal MRI and BLI data set from one animal that received MCAO surgery. MRI is shown as coronal brain section, BLI is shown as horizontal planar projection of the PE onto the mouse photograph. After MCAO, a clear lesion is visible in the right hemisphere on T2 maps. BLI signal intensity starts to increase 3d post-stroke and PE is clearly increased over the ischemic hemisphere compared to the intact hemisphere at 7d and 14d post-stroke. Increased PE from the ischemic hemisphere is distinctively visualized in the mirrors. **(B)** Representative longitudinal MRI and BLI data set from one animal that received sham surgery. Sham surgery did not result in lesion formation confirmed by unchanged T2 maps. However, sham surgery lead to a transient increase in PE from the ipsilateral side, which was best visible in the mirror system. (**C)** BLI kinetics of the ischemic and the intact hemisphere at 14d post sham surgery (upper graph) and MCAO surgery (lower graph). **(D)** Quantification of BLI changes was achieved by normalization of PE from ipsi- to contralateral hemisphere for the top view (left graph) and the mirror view (right graph). Pre-stroke PE was consistently equal in all investigated groups with a stronger variation in the mirror view. Increased PE was significantly different to pre-stroke values on day three for stroke, but also for sham animals in both views. At 7d and 14d, elevated PE in stroke animals was statistically significant compared to pre-stroke values (both *p* < 0.001) and to the sham group (*p* = 0.001, *p* = 0.004). (black = stroke, gray = sham, * *p* < 0.05, ** *p* < 0.01, *** *p* < 0.001; # comparison between groups *p* < 0.01).

Cerebral ischemia was induced by 30 min occlusion of the right MCA with a silicone rubber-coated filament. Lesion size, location and development were assessed by MRI using quantitative T2 maps. Lesions appear as areas of increased T2 values on T2 maps, and a representative lesion is displayed in Figure 3A. Lesions were of similar size throughout all groups, including damage in the striatum and the parietal cortex, except for one animal of the 7d IHC group that showed only a small striatal infarct. At 14 days after MCAO, the ischemic hemisphere has shrunken in size, giving space to cerebrospinal fluid, which is visible as a rim of increased T2 value along the ischemic cortex.

### Bioluminescence of VEGFR2 expression after stroke

Before MCAO, T2 maps displayed no signs of lesion and PE was comparable for the right and left hemisphere (Figure 3A, first row). Three days after MCAO, vasogenic edema resulted in increased T2 values, and a clear lesion was visible in the right hemisphere on T2 maps (Figure 3A, second row). Middle cerebral artery occlusion induced a significant increase in PE from the ischemic hemisphere (top view: *F*_(1.8, 33.7)_ = 14.8, *p* < 0.001; mirror view: *F*_(3, 45)_ = 12.224, *p* < 0.001). Photon emission from the ischemic hemisphere was already significantly increased at 3 days post MCAO compared to pre stroke values (top view: *p* = 0.002, mirror view: *p* = 0.006) indicating an early upregulation of VEGFR2 expression. Photon emission continued to increase from the ischemic hemisphere and was clearly visible on BL images on 7d and 14d after MCAO. The maximal PE increase of 58 ± 36% (*p* = < 0.001) in the ischemic hemisphere was observed 7d post MCAO in the top view (Figure 3D, left graph). The PE increase was even more pronounced (78 ± 26%; *p* < 0.001) when observed through the mirror system (Figure 3D, right graph). At the last observation time point of 14d post MCAO, the PE was still strongly increased in the ischemic hemisphere in both, the top view (44 ± 34%; *p* = 0.001) and the mirror view (65 ± 26%; *p* = 0.001). The pathological condition of ischemia does not alter the BLI kinetics described for healthy control mice. Photon emission from the ischemic hemisphere reaches the plateau phase also between 10–13 min but with higher PE than from the intact hemisphere (Figure 3C).

Sham surgery (introduction of the filament into the ICA but without advancing it to occlude the MCA) did not result in lesion formation (cf. T2 maps in Figure 3B), but, nevertheless, resulted in a transient change in PE on the ispilateral hemisphere (Figures 3B,D) at 3 days after surgery (top view: 15 ± 18% *p* = 0.019; mirror view: 58 ± 31% *p* = 0.026). The increase in emission was of similar magnitude as observed in the MCAO group. Although emission from the sham hemisphere stayed elevated at 7d and 14d, the change to the pre-surgery values was no longer significant (Figure 3D). The upregulation of VEGFR2 expression was significantly higher in MCAO animals when compared to sham animals (*F*_(1.77, 33.67)_ = 10.19 *p* = 0.001) at 7d (*p* = 0.001) and at 14d (*p* = 0.004).

### Western blot analysis

**Figure 4:**
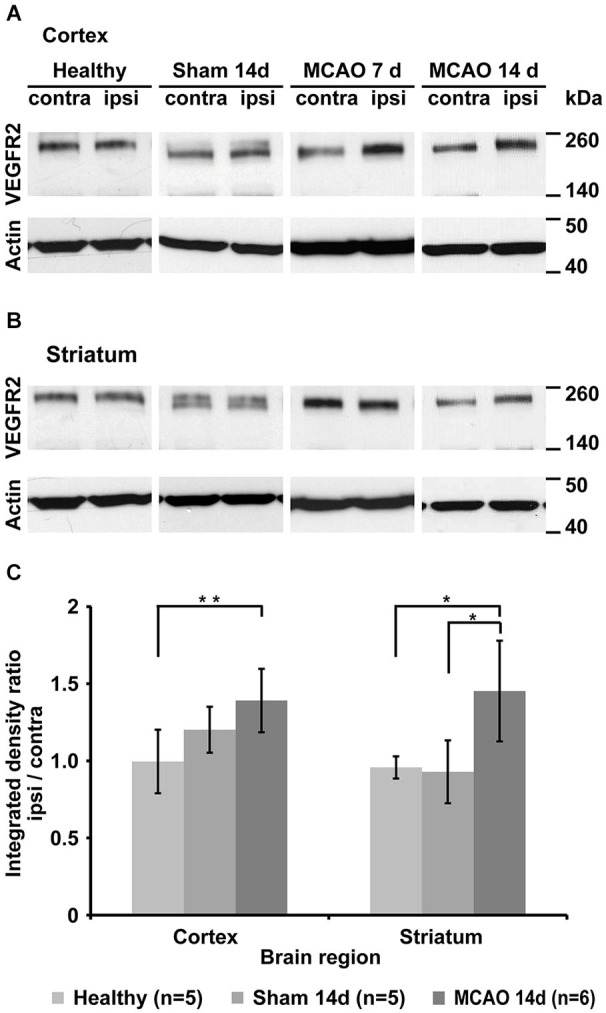
**Qualitative and quantitative evaluation of tissue VEGFR2 protein content. (A)** Representative Western blots from cortical tissue samples for each group showing increased VEGFR2 (210 and 230 kDa) content in the ischemic cortex of animals that underwent MCAO. Note the strong increase in the 7d group. Sham animals and healthy control display similar levels between left and right hemisphere. **(B)** Representative Western blots from striatal tissue samples. Strongest elevation is visible in the 14d MCAO group. **(C)** Semi-quantification (including normalization to actin) and subsequent normalization to the intact hemisphere reveals significant increased VEGFR2 expression in the ischemic cortex (*p* = 0.005) and ischemic striatum (*p* = 0.048) (* *p* < 0.05, ** *p* < 0.01).

Western blot analysis of healthy control animals shows little change in VEGFR2 expression between left and right hemisphere. Middle cerebral artery occlusion results in changes in VEGFR2 protein content of the ischemic hemisphere (Figures 4A,B). Significantly elevated levels of VEGFR2 protein were found in the ischemic cortex (40 ± 21%, *p* = 0.005) and in the ischemic striatum (32 ± 43%, *p* = 0.048) compared to healthy control animals (Figure 4C). Sham surgery resulted in non-significant elevation of VEGFR2 protein in the cortex (20 ± 15%), but an apparent decrease in the striatum (−7 ± 20%; Figure 4C).

### Immunohistochemistry

**Figure 5:**
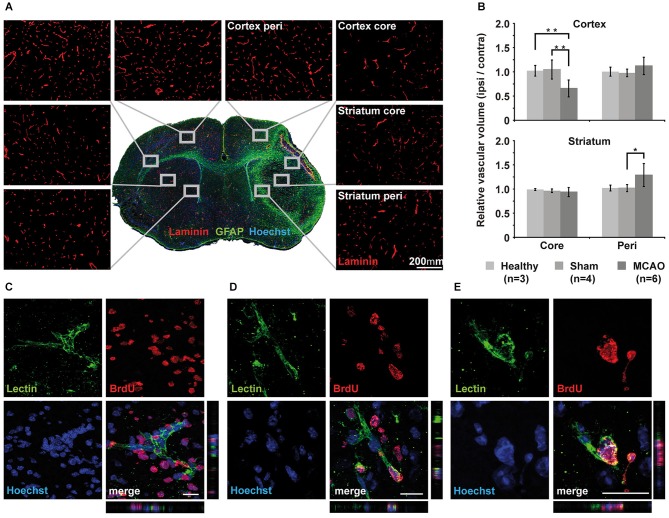
**Immunohistochemical analysis of vascular changes. (A)** Immunohistochemistry of laminin for vascular volume estimation. Representative close-ups of 20x magnification for each region of interest visualize changes in vessel density. **(B)** Quantification and subsequent normalization to the intact hemisphere confirms decreased vascular volume in the core region of the cortex but not in the core region of the striatum. Vascular volume increases significantly in the striatal peri-infarct zone (*p* = 0.03) but does not reach significance in peri-infarct cortex (*p* = 0.08). Sham animals showed little changes in corresponding areas of the brain. **(C–E)** Three examples of Z-stacks of representative BrdU+/lectin+newly formed endothelial cells from the peri-infarct striatum **(C)** and the cortex **(D,E)**. Scale bar 20 μm.

Histology was performed on the brains to determine changes in vascular volume after MCAO. 10-μm-thick brain sections were therefore stained with laminin. The stained area was interpreted as vascular volume. Glial fibrillary acidic protein staining was used for characterization of astrocyte activation in association with the ischemic lesion. Overview images were acquired for identification of the four areas of interest: the core lesion in the cortex (cortex core), the peri-infarct area of the cortical lesion (cortex peri), the striatal core lesion (striatum core), and the peri-infarct area of the striatal lesion (striatum peri). Three close-ups of 20× magnification were taken from each area of interest and from corresponding sites of the intact hemisphere with equal exposure times. Figure 5A shows representative close-ups of each region. As a measure for vascular volume changes, the area positive for laminin was compared between the ischemic hemisphere and the corresponding regions on the contralateral hemisphere (% area, normalized to contralateral side). Vascular volume was strongly reduced in the cortical core region in MCAO animals at 14 days after stroke (34 ± 17%, *p* = 0.08), but not in the striatal core. Peri-infarct regions of both, cortex and striatum, showed increased vascular volume, reaching significance in the striatum (29 ± 23%, *p* = 0.03; Figure 5B). Sham animals did not differ from healthy animals in all investigated brain regions. In each region that showed increased vascular volume, we observed endothelial cells with incorporated BrdU (Figures 5C–E), indicating that angiogenic signaling resulted in endothelial cell proliferation during day 3 and 7 after MCAO.

### Correlation of bioluminescence intensity with protein expression and vascular volume

**Figure 6:**
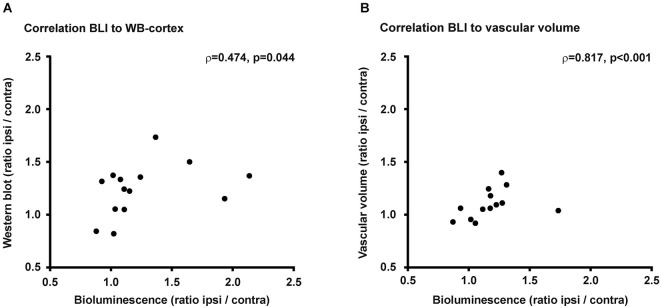
**Correlations. (A)** Changes of PE within the ischemic hemisphere correlates to VEGFR2 protein changes in the ischemic cortex (*ρ* = 0.474, *p* = 0.044). **(B)** Photon emission increase also correlates to vascular volume changes within the ischemic hemisphere (*ρ* = 0.817, *p* < 0.001).

Middle cerebral artery occlusion resulted in an increase in bioluminescene intensity from the ischemic hemisphere, which was paralleled by the observation of increased VEGFR2 protein content and increased vascular volume. For the completeness of this study we performed correlation analysis of the bioluminescence signal intensity with VEGFR2 protein content on the one side, or with vascular volume on the other side (Figure 6). Bioluminescence intensity correlates well with VEGFR2 protein content in the cortex (*ρ* = 0.474, *p* = 0.44). No correlation was found between bioluminescence and protein content in the striatum, which is due to the stronger absorption of photons from this deeper structure. Therefore signal from the striatum will be overlayed by signal from the cortex. Also a significant correlation was observed to vascular volume in peri-infarct areas (*ρ* = 0.817, *p* < 001).

## Discussion

We non-invasively observed the temporal profile of VEGFR2 expression after cerebral ischemia as a molecular reporter for post-stroke pro-angiogenic signaling, while we followed lesion development with the complementary method of MRI. VEGFR2 plays a key role in post-ischemic vascular remodeling and we found increased expression lasting up to 14d post MCAO, which was paralleled by an increased vascular volume in distinct peri-infarct areas.

### Methodological considerations

Bioluminescence imaging is a very sensitive method and allows for the detection of even small changes in cell numbers (Keyaerts et al., 2008). However, it has a poor spatial resolution. For the first time we report here the additional observation of PE through two 45° angled mirrors for a better discrimination of left and right hemisphere through lateral views. Signal changes were consistently greater with intensity readouts from the mirror views, but variation of the signal was also increased, suggesting the introduction of additional noise. *In vitro*, a linear correlation exists between PE and number of luciferase expressing cells. *In vivo* this relation is affected by luciferin distribution and photon absorption and scattering by tissue (Virostko et al., 2004; Inoue et al., 2009; Keyaerts et al., 2011). Bioluminence imaging of the brain has to deal with limited substrate diffusibility through the blood brain-barrier (BBB; Berger et al., 2008). Brain pathologies, which result in a breakdown of the BBB, impose even further methodological obstacles towards the interpretation of the PE. Theoretically, an open BBB may facilitate luciferin inflow on the ischemic hemisphere, thus resulting in higher PE due to higher substrate availability, possibly mistaken for higher luciferase content. However, we can exclude the open BBB as dominating confounding factor in this animal model and study, since sham animals with intact BBB have PE increase equal to MCAO animals with open BBB at 3d post surgery. If carotid artery occlusion (sham surgery) disturbs the BBB integrity, it will be of much less intensity than by stroke. Hence, if the increase at that time point was only due to increased luciferin content rather than increased expression of VEGFR2 (and therefore luciferase), then we should have had observed stronger signal in MCAO compared to sham. Furthermore, increased wash-in of luciferin due to an open BBB would result in faster slopes of the signal kinetics. This, however, was not seen at all. We can therefore safely conclude that BBB breakdown in this model does not affect the bioluminescence reaction and that the increase in PE can be solely attributed to increased VEGFR2 expression.

### VEGFR2 upregulation during post-stroke angiogenesis

In order to non-invasively monitor VEGFR2 expression following stroke, we made use of a transgenic mouse expressing luciferase under the control of the VEGFR2 promotor (Lyons et al., 2005). We observed increasing bioluminescence intensity after cerebral ischemia in the ischemic hemisphere, which peaked at 7d post-stroke. In agreement with previous reports (Marti et al., 2000; Hayashi et al., 2003; Cai et al., 2009), our results show a continuous increase in VEGFR2 expression from 3d to 7d post-stroke. We observe still significantly elevated expression at 14d post-stroke, which was confirmed by increased VEGFR2 protein content in the ischemic hemisphere compared to the intact hemisphere. Semi-quantitative analysis of regional VEGFR2 content in the brain by Western blotting indicates strong expression in the ischemic striatum and cortex, since the amount of VEGFR2 was increased by as much as 40%. Also sham surgery resulted in a transient increase in BLI signal from the sham hemisphere, indicating also upregulated VEGFR2 expression. Western blot results show a slightly increased VEGFR2 content at 14d post surgery in the right cortex, but not in the striatum of sham animals. Sham surgery involved the introduction of a filament only into the CCA. Upon removal of the filament, the CCA was ligated permanently to control for blood flow changes induced by the permanent ligation of the CCA in the stroke group. Common carotid artery occlusion is used as a model for mild hypoxia and chronic cerebral hypoperfusion (Hecht et al., 2012; Pimentel-Coelho et al., 2012). Following the occlusion of the ICA, Hecht et al. (2012) observed reduction by 80% in overall cerebral blood flow, while cortical perfusion was not notably changed. Unilateral CCA occlusion did not result in neuronal cell death within the territory of the MCAO (Pimentel-Coelho et al., 2012), but at 21d after ICA occlusion, slightly increased vessel density was noted in the ipsilateral cortex (Hecht et al., 2012). Although our study did not indicate a strong increase in cortical or striatal vascular volume, BLI was sensitive enough to detect the minor changes in VEGFR2 expression induced by mild hypoxia after CCA occlusion.

### Vascular volume increase in peri-infarct areas

We investigated the vascular volume in core regions and in peri-infarct regions of both, the cortex and the striatum. Our MCAO model resulted in strong reduction in vascular volume within the cortical core region. Similar observations were made by Bosomtwi et al. (2011) in a distal MCAO rat model where only the cortex is affected. Yet, in the region defined as striatum core, we detected no decrease in vessel density. High upregulation of VEGFR2 in the striatum, as indicated by our WB results, may account for early enhanced endothelial survival due to protective properties of VEGF2 (Ferrara, 2004; Shibuya, 2006; Lee et al., 2007a; Hermann and Zechariah, 2009), and may even have been followed by stabilization of existing or recruitment of new vessels. In accordance with previous studies (Marti et al., 2000; Hayashi et al., 2003; Thored et al., 2007; Li et al., 2011; Reitmeir et al., 2012), we observed increased vascular volume in the peri-infarct striatum (29 ± 23%) and peri-infarct cortex (12% ± 17%). Vascular volume increase in the striatal peri-infarct was most pronounced in the most dorsal part of the striatum close to the subventricular zone (SVZ), which has been described as highly angiogenic (Ohab et al., 2006; Thored et al., 2007). Strong endothelial cell proliferation had been observed in this area next to the SVZ between 1 and 2 weeks, and consequently increased vessel density persisted up to 16 weeks (Thored et al., 2007). Other studies reported endothelial cell proliferation starting as early as 24 h after MCAO in mice (Hayashi et al., 2003), and increased vessel density was detectable at 2–3d post-stroke (Marti et al., 2000; Hayashi et al., 2003). The number of microvessels remained increased up to 21d (Hayashi et al., 2003). In both regions with increased vascular volume, striatal and cortical peri-infarct, we detected proliferating endothelial cells, which were newly generated in the first week after stroke, due to BrdU incorporation. As new vessels can also form without endothelial cell proliferation by splitting existing vessels through the insertion of tissue pillars, intussusception (Adams and Alitalo, 2007) and peripheral bone marrow-derived endothelial progenitor cells can contribute to vessel formation or stabilization (Zhang et al., 2002) we restrained from quantification of BrdU positive endothelial cells as this would underestimate the intensity of increased vascular volume.

### Stroke-induced angiogenic signaling can be non-invasively quantified with the VEGFR2-luc mouse model

The bioluminescence signal change reflected increased VEGFR2 expression within the ischemic hemisphere. Western blot analysis revealed increased VEGFR2 protein concentration in both, ischemic striatum and cortex. In both regions, increased VEGFR2 expression translated into increased vascular volume. A positive correlation, not very strong but significant, between bioluminescence intensity and VEGFR2 protein content within the cortex was observed and renders this mouse model an optimal model for non-invasive tracking of VEGFR2 regulation in the brain. No correlation was observed to VEGFR2 content of the striatum. As BLI signal recorded by the CCD camera is a two-dimensional image from the three-dimensional structure of the brain, signals from deeper structures like the striatum are subjected to stronger absorption by overlaying tissue than signals from structures closer to the brain surface like the cortex. Hence, the detected bioluminescence signal in this model is strongly dominated by its contribution from cortex. A significant correlation was also detected between PE intensity and vascular volume. Such a correlation was not expected because of the time delay between VEGFR2 expression and the occurrence of laminated microvessels, and due to additional expression of VEGFR2 by cells other than proliferating endothelial cells (Hayashi et al., 2003). VEGFR2 is mainly expressed on endothelial cells and functions as transducer of survival, proliferation, migration and differentiation cues (Ferrara et al., 2003). Following stroke, the number of VEGFR2 positive endothelial cells was strongly increased in the penumbra (Li et al., 2011), but also post-ischemic neurons started to express this receptor (Hayashi et al., 2003; Beck and Plate, 2009). However, neuronal VEGFR2 expression was an early and transient effect, declining already 3 days after ischemia, while its expression remained strong in endothelial cells (Hayashi et al., 2003). Vascular endothelial growth factor receptor 2 expression was also reported on astrocytes (Issa et al., 1999) and microglia/macrophages (Lennmyr et al., 1998) following stroke, however, other groups could not reproduce these results (Krum et al., 2002). Further, a distinct pattern, where astrocytes express VEGFR1 and endothelial cells express VEGFR2 was suggested (Krum et al., 2008). Therefore, we believe it is justified to neglect the effect of neuronal and glial poststroke VEGFR2 in our study. The changes in VEGFR2 levels we observe in the chronic phase after stroke represent predominantly changes of VEGFR2 on endothelial cells. New vessel formation is not restricted to on-site endothelial cell proliferation but can occur also by intussusception (Adams and Alitalo, 2007) and by the contribution of peripheral bone marrow-derived endothelial progenitor cells (Zhang et al., 2002). Also in these processes signaling via the VEGFR2 plays a pivotal role. Poststroke angiogenesis is VEGF/VEGFR2 signaling dependent, as selectively blocking the VEGFR2 leads to decreased endothelial cell proliferation and reduced cerebral blood flow (Li et al., 2011). We can summarize that pro-angiogenic signaling via the VEGF—VEGFR2 pathway is a general necessity for new vessel formation, and non-invasive observation of the VEGFR2 expression provides longitudinal information about poststroke neurovascular dynamics.

## Conclusion

The present study established the value of the VEGFR2-luc mouse model for non-invasive tracking of VEGFR2 expression as a correlate for neurovascular remodeling in stroke pathology. We have deciphered non-invasively and quantitatively a temporal profile of VEGFR2 expression for the first 2 weeks after stroke. Also, we have observed, that chronic VEGFR2 expression correlates well with vascular volume increase in cortical peri-infarct regions. Future studies will benefit from this newly validated non-invasive tool for investigations of angiogenesis promoting therapies.

## Conflict of interest statement

The authors declare that the research was conducted in the absence of any commercial or financial relationships that could be construed as a potential conflict of interest.

## Footnotes

^1^http://rsbweb.nih.gov/ij/

## References

[B1] Adams R. H. Alitalo K. (2007). Molecular regulation of angiogenesis and lymphangiogenesis. Nat. Rev. Mol. Cell Biol. 8, 464–478 10.1038/nrm2183 17522591

[B2] Bahmani P. Schellenberger E. Klohs J. Steinbrink J. Cordell R. Zille M. (2011). Visualization of cell death in mice with focal cerebral ischemia using fluorescent annexin A5, propidium iodide and TUNEL staining. J. Cereb. Blood Flow Metab. 31, 1311–1320 10.1038/jcbfm.2010.233 21245871 PMC3099638

[B3] Beck H. Acker T. Wiessner C. Allegrini P. R. Plate K. H. (2000). Expression of angiopoietin-1, angiopoietin-2 and tie receptors after middle cerebral artery occlusion in the rat. Am. J. Pathol. 157, 1473–1483 10.1016/s0002-9440(10)64786-4 11073808 PMC1885747

[B4] Beck H. Plate K. H. (2009). Angiogenesis after cerebral ischemia. Acta Neuropathol. 117, 481–496 10.1007/s00401-009-0483-6 19142647

[B5] Berger F. Paulmurugan R. Bhaumik S. Gambhir S. S. (2008). Uptake kinetics and biodistribution of 14C-D-luciferin—a radiolabeled substrate for the firefly luciferase catalyzed bioluminescence reaction: impact on bioluminescence based reporter gene imaging. Eur. J. Nucl. Med. Mol. Imaging 35, 2275–2285 10.1007/s00259-008-0870-6 18661130 PMC4157642

[B6] Bosomtwi A. Chopp M. Zhang L. Zhang Z. G. Lu M. Jiang Q. (2011). Mean microvessel segment length and radius after embolic stroke: comparison of magnetic resonance imaging (MRI) and laser scanning confocal microscopy (LSCM). Brain Res. 1381, 217–227 10.1016/j.brainres.2011.01.009 21237138 PMC3056053

[B7] Cai W. Guzman R. Hsu A. R. Wang H. Chen K. Sun G. (2009). Positron emission tomography imaging of poststroke angiogenesis. Stroke 40, 270–277 10.1161/STROKEAHA.108.517474 18948613

[B8] Ferrara N. (2004). Vascular endothelial growth factor: basic science and clinical progress. Endocr. Rev. 25, 581–611 10.1210/er.2003-0027 15294883

[B9] Ferrara N. Gerber H. P. LeCouter J. (2003). The biology of VEGF and its receptors. Nat. Med. 9, 669–676 10.1038/nm0603-669 12778165

[B10] Hayashi T. Deguchi K. Nagotani S. Zhang H. Sehara Y. Tsuchiya A. (2006). Cerebral ischemia and angiogenesis. Curr. Neurovasc. Res. 3, 119–129 10.2174/156720206776875902 16719795

[B11] Hayashi T. Noshita N. Sugawara T. Chan P. H. (2003). Temporal profile of angiogenesis and expression of related genes in the brain after ischemia. J. Cereb. Blood Flow Metab. 23, 166–180 10.1097/00004647-200302000-00004 12571448

[B12] Hecht N. He J. Kremenetskaia I. Nieminen M. Vajkoczy P. Woitzik J. (2012). Cerebral hemodynamic reserve and vascular remodeling in C57/BL6 mice are influenced by age. Stroke 43, 3052–3062 10.1161/STROKEAHA.112.653204 22923448

[B13] Hermann D. M. Zechariah A. (2009). Implications of vascular endothelial growth factor for postischemic neurovascular remodeling. J. Cereb. Blood Flow Metab. 29, 1620–1643 10.1038/jcbfm.2009.100 19654590

[B14] Inoue Y. Kiryu S. Izawa K. Watanabe M. Tojo A. Ohtomo K. (2009). Comparison of subcutaneous and intraperitoneal injection of D-luciferin for in vivo bioluminescence imaging. Eur. J. Nucl. Med. Mol. Imaging 36, 771–779 10.1007/s00259-008-1022-8 19096841

[B15] Issa R. Krupinski J. Bujny T. Kumar S. Kaluza J. Kumar P. (1999). Vascular endothelial growth factor and its receptor, KDR, in human brain tissue after ischemic stroke. Lab. Invest. 79, 417–425 10211994

[B16] Keyaerts M. Heneweer C. Gainkam L. O. Caveliers V. Beattie B. J. Martens G. A. (2011). Plasma protein binding of luciferase substrates influences sensitivity and accuracy of bioluminescence imaging. Mol. Imaging Biol. 13, 59–66 10.1007/s11307-010-0325-x 20383591

[B17] Keyaerts M. Verschueren J. Bos T. J. Tchouate-Gainkam L. O. Peleman C. Breckpot K. (2008). Dynamic bioluminescence imaging for quantitative tumour burden assessment using IV or IP administration of D: -luciferin: effect on intensity, time kinetics and repeatability of photon emission. Eur. J. Nucl. Med. Mol. Imaging 35, 999–1007 10.1007/s00259-007-0664-2 18180921

[B18] Koch S. Tugues S. Li X. Gualandi L. Claesson-Welsh L. (2011). Signal transduction by vascular endothelial growth factor receptors. Biochem. J. 437, 169–183 10.1042/BJ20110301 21711246

[B19] Krum J. M. Mani N. Rosenstein J. M. (2002). Angiogenic and astroglial responses to vascular endothelial growth factor administration in adult rat brain. Neuroscience 110, 589–604 10.1016/s0306-4522(01)00615-7 11934468

[B20] Krum J. M. Mani N. Rosenstein J. M. (2008). Roles of the endogenous VEGF receptors flt-1 and flk-1 in astroglial and vascular remodeling after brain injury. Exp. Neurol. 212, 108–117 10.1016/j.expneurol.2008.03.019 18482723 PMC2478519

[B21] Krupinski J. Kaluza J. Kumar P. Kumar S. Wang J. M. (1994). Role of angiogenesis in patients with cerebral ischemic stroke. Stroke 25, 1794–1798 10.1161/01.str.25.9.1794 7521076

[B22] Lee S. Chen T. T. Barber C. L. Jordan M. C. Murdock J. Desai S. (2007a). Autocrine VEGF signaling is required for vascular homeostasis. Cell 130, 691–703 10.1016/j.cell.2007.06.054 17719546 PMC3010851

[B23] Lee H. J. Kim K. S. Park I. H. Kim S. U. (2007b). Human neural stem cells over-expressing VEGF provide neuroprotection, angiogenesis and functional recovery in mouse stroke model. PLoS One 2:e156 10.1371/journal.pone.0000156 17225860 PMC1764718

[B24] Lennmyr F. Ata K. A. Funa K. Olsson Y. Terent A. (1998). Expression of vascular endothelial growth factor (VEGF) and its receptors (Flt-1 and Flk-1) following permanent and transient occlusion of the middle cerebral artery in the rat. J. Neuropathol. Exp. Neurol. 57, 874–882 10.1097/00005072-199809000-00009 9737551

[B25] Li W. L. Fraser J. L. Yu S. P. Zhu J. Jiang Y. J. Wei L. (2011). The role of VEGF/VEGFR2 signaling in peripheral stimulation-induced cerebral neurovascular regeneration after ischemic stroke in mice. Exp. Brain Res. 214, 503–513 10.1007/s00221-011-2849-y 21922279

[B26] Lyons S. K. Clermont A. O. Neben T. Y. Campbell K. Coffee R. Hunter J. (2005). Non-invasive in vivo bioluminescent imaging of tumor angiogenesis in mice. AACR Meeting Abstracts 2005, 903-b

[B27] Marti H. J. Bernaudin M. Bellail A. Schoch H. Euler M. Petit E. (2000). Hypoxia-induced vascular endothelial growth factor expression precedes neovascularization after cerebral ischemia. Am. J. Pathol. 156, 965–976 10.1016/s0002-9440(10)64964-4 10702412 PMC1876841

[B28] Navaratna D. Guo S. Arai K. Lo E. H. (2009). Mechanisms and targets for angiogenic therapy after stroke. Cell Adh. Migr. 3, 216–223 10.4161/cam.3.2.8396 19363301 PMC2679890

[B29] Ohab J. J. Fleming S. Blesch A. Carmichael S. T. (2006). A neurovascular niche for neurogenesis after stroke. J. Neurosci. 26, 13007–13016 10.1523/jneurosci.4323-06.2006 17167090 PMC6674957

[B30] Pimentel-Coelho P. M. Michaud J. P. Rivest S. (2012). Effects of mild chronic cerebral hypoperfusion and early amyloid pathology on spatial learning and the cellular innate immune response in mice. Neurobiol. Aging 34, 679–693 10.1016/j.neurobiolaging.2012.06.025 22819135

[B31] Reitmeir R. Kilic E. Reinboth B. S. Guo Z. Elali A. Zechariah A. (2012). Vascular endothelial growth factor induces contralesional corticobulbar plasticity and functional neurological recovery in the ischemic brain. Acta Neuropathol. 123, 273–284 10.1007/s00401-011-0914-z 22109109

[B32] Shibuya M. (2006). Differential roles of vascular endothelial growth factor receptor-1 and receptor-2 in angiogenesis. J. Biochem. Mol. Biol. 39, 469–478 10.5483/bmbrep.2006.39.5.469 17002866

[B33] Shibuya M. (2009). Brain angiogenesis in developmental and pathological processes: therapeutic aspects of vascular endothelial growth factor. FEBS J. 276, 4636–4643 10.1111/j.1742-4658.2009.07175.x 19664071

[B34] Slevin M. Kumar P. Gaffney J. Kumar S. Krupinski J. (2006). Can angiogenesis be exploited to improve stroke outcome? Mechanisms and therapeutic potential. Clin. Sci. (Lond). 111, 171–183 10.1042/cs20060049 16901264

[B35] Szpak G. M. Lechowicz W. Lewandowska E. Bertrand E. Wierzba-Bobrowicz T. Dymecki J. (1999). Border zone neovascularization in cerebral ischemic infarct. Folia Neuropathol. 37, 264–268 10705649

[B36] Taguchi A. Soma T. Tanaka H. Kanda T. Nishimura H. Yoshikawa H. (2004). Administration of CD34+ cells after stroke enhances neurogenesis via angiogenesis in a mouse model. J. Clin. Invest. 114, 330–338 10.1172/jci200420622 15286799 PMC484977

[B37] Thored P. Wood J. Arvidsson A. Cammenga J. Kokaia Z. Lindvall O. (2007). Long-term neuroblast migration along blood vessels in an area with transient angiogenesis and increased vascularization after stroke. Stroke 38, 3032–3039 10.1161/strokeaha.107.488445 17901386

[B38] Virostko J. Chen Z. Fowler M. Poffenberger G. Powers A. C. Jansen E. D. (2004). Factors influencing quantification of in vivo bioluminescence imaging: application to assessment of pancreatic islet transplants. Mol. Imaging 3, 333–342 10.1162/1535350042973508 15802050

[B39] Wang Y. Galvan V. Gorostiza O. Ataie M. Jin K. Greenberg D. A. (2006). Vascular endothelial growth factor improves recovery of sensorimotor and cognitive deficits after focal cerebral ischemia in the rat. Brain Res. 1115, 186–193 10.1016/j.brainres.2006.07.060 16928361

[B40] Zhang Z. G. Zhang L. Jiang Q. Chopp M. (2002). Bone marrow-derived endothelial progenitor cells participate in cerebral neovascularization after focal cerebral ischemia in the adult mouse. Circ. Res. 90, 284–288 10.1161/hh0302.104460 11861416

